# Bumblebees require visual pollen stimuli to initiate and multimodal stimuli to complete a full behavioral sequence in close‐range flower orientation

**DOI:** 10.1002/ece3.2768

**Published:** 2017-02-01

**Authors:** Saskia Wilmsen, Robin Gottlieb, Robert R. Junker, Klaus Lunau

**Affiliations:** ^1^ Department BiologyInstitute of Sensory EcologyHeinrich‐Heine‐UniversityDüsseldorfGermany; ^2^ Department of Ecology and EvolutionUniversity of SalzburgSalzburgAustria

**Keywords:** *Bombus terrestris*, bumblebee, flower recognition, multimodal stimuli, pollen, stamen mimicry

## Abstract

Flower visits are complex encounters, in which animals are attracted by floral signals, guided toward the site of the first physical contact with a flower, land, and finally take up floral rewards. At close range, signals of stamens and pollen play an important role to facilitate flower handling in bees, yet the pollen stimuli eliciting behavioral responses are poorly known. In this study, we test the response of flower‐naive bumblebees (*Bombus terrestris*) toward single and multimodal pollen stimuli as compared to natural dandelion pollen. As artificial pollen stimuli, we used the yellow flavonoid pigment quercetin, the scent compound eugenol, the amino acid proline, the monosaccharide glucose, and the texture of pollen‐grain‐sized glass pellets as a tactile stimulus. Three test stimuli, dandelion pollen, one out of various uni‐ and multimodal stimulus combinations, and a solvent control were presented simultaneously to individual bumblebees, whose response was recorded. The results indicate that bumblebees respond in an irreversible sequence of behavioral reactions. Bumblebees approached the visual stimulus quercetin as often as natural dandelion pollen. An additional olfactory stimulus resulted in slightly more frequent landings. The multimodal stimulus combinations including visual, olfactory, gustatory, and tactile stimuli elicited approaches, antennal contacts, and landings as often as natural pollen. Subsequent reactions like proboscis extension, mandible biting, and buzzing were more often but not regularly observed at dandelion pollen. Our study shows that visual signals of pollen are sufficient to trigger initial responses of bumblebees, whereas multimodal pollen stimuli elicit full behavioral response as compared to natural pollen. Our results suggest a major role of pollen cues for the attraction of bees toward flowers and also explain, why many floral guides mimic the visual signals of pollen and anthers, that is, the yellow and UV‐absorbing color, to direct bumblebees toward the site where they access the floral rewards.

## 1 Introduction

Many flowering plants depend on insects as pollinators to secure reproduction, while offering nectar, pollen, and other resources as primary rewards. Flowers are sensory billboards (Raguso, 2004) displaying multimodal signals (Junker & Parachnowitsch, 2015) including visual signals, for example, shape, symmetry, color patterns, colors and contrasts as visual signals (Dafni & Kevan, 1996; Giurfa et al., 1999; Lehrer, Horridge, Zhang, & Gadagkar, 1995; Lunau, 2011; Osche, 1983), olfactory signals (Raguso, 2009), tactile cues (Kevan & Lane, 1985), and gustatory stimuli (Lipp, 1991; Willmer, 2011). Flowers attract pollinators from a distance, guide them at close range toward a distinct site of the flower, trigger the landing on the flower, and facilitate finding the floral resource while transferring pollen from the pollinator to the stigma and from the flower to the pollinators’ body (Lunau, 1991, 1992). Whereas colored bracts or petals are mostly large‐sized to attract flower visitors from some distance, color patterns of the petals and other flower organs are small and more important for orientation at close range. This visual color pattern is paralleled by an olfactory, gustatory and tactile pattern (Burdon, Raguso, Kessler, & Parachnowitsch, 2015). Studies of flower odor chemistry suggest that nectar and pollen rewards frequently differ in the quantity and composition of volatile compounds compared to other floral tissues, and that such within‐flower spatial variation may function as odor guides to direct pollinators to nectar (Dötterl & Jürgens, 2005) or pollen rewards (Bergström et al., 1995; Dobson, Danielson, & Van Wesep, 1999). Stamens, that is, anthers and pollen, take over important parts of floral signaling (Lunau, 2000) when offering visually, chemically and tactily conspicuous pollen that many flower visitors can eat or collect.

Female bees need at least two floral resources, nectar for individual energy supply and pollen for egg maturation (Cane, 2016) and the provision of their offspring (Alford, 1975; Roulston & Cane, 2000). The bees’ response to various stimuli associated with nectar including color (Zhang, Larson‐Rabin, Li, & Wang, 2012), glistening (Endress & Steiner‐Gafner, 1996), scent (Howell & Alarcón, 2007), and taste (Frost, Shutler, & Hillier, 2012; Gil, Menzel, & De Marco, 2008; Haupt, 2004; Menzel, 1993) have been extensively studied, whereas similar responses to pollen are quite little studied. Contrary to nectar, remote sensing of pollen by means of visual signals, mostly caused by yellow pigments, are important cues for pollen‐eating and pollen‐collecting insects (Lunau, 2000, 2006). Comparable to nectar, pollen contains chemical cues emitted from the pollenkitt of bee‐pollinated flowers (Dobson, 1988; Dobson & Bergström, 2000; Stanley & Linskens, 1974), but their role for identification of pollen as such is not known. Pollenkitt is a sticky pollen coat material produced by the anther tapetum of most angiosperms (Pacini & Hesse, 2005) and produces the relevant visual and chemical cues of pollen for pollinators. Differently from nectar, it seems impossible for bees to sense the relevant nutrients of pollen, proteins, until the pollen grains have been crushed with the mandibles (Lunau, Piorek, Krohn, & Pacini, 2015). Moreover, sensing of proteins by means of insect taste receptors is not known (De Brito Sanchez, 2011; Rüdenauer, Späthe, & Leonhardt, 2015).

It is known that bumblebees exhibit a specific behavior to visual signals of pollen that enable them to subsequently check chemical and tactile signals (Lunau, 1991, 1992; Lunau, Fieselmann, Heuschen, & van de Loo, 2006). In a visually guided approach, naive bumblebees target yellow‐colored pollen signals and precisely make contact with these signals with the tips of their antennae (Pohl & Lunau, 2007). Once the antennae have contact with the object, chemical and tactile stimuli gain significance (Goulson, 2003; De Brito Sanchez, 2011). Beside tarsi, mandibles, proboscis, and thorax, the antennae tips contain the largest number of sensory cells (Ägren & Hallberg, 1996; De Brito Sanchez et al., 2008). These sensilla provide the reception of temperature and humidity (Ägren & Hallberg, 1996) as well as olfactory, gustatory, and mechanoreceptive input (Dietz & Humphreys, 1971).

Thus, it remains unknown how bees detect and identify pollen. In order to test the significance of single and multiple stimuli of pollen for their impact on behavioral reactions in workers of the buff‐tailed bumblebee, *Bombus terrestris*, we used a triple choice experiment. Next to each other we presented three target objects, hand‐collected pure dandelion pollen (*Taraxacum officinale*), a unimodal or multimodal pollen stimulus combination, and a solvent control.

## 2 Material and Methods

### 2.1 Experimental setup

Our experimental approach focuses on the hypothetical function of visual, olfactory, gustatory, and tactile stimuli of pollen grains for bees and likewise a hypothetical synergistic effect of multimodal pollen stimuli (Junker & Parachnowitsch, 2015; Junker et al., 2013; Katzenberger, Lunau, & Junker, 2013; Leonard & Masek, 2014). The rationale of this approach is based on the knowledge about typical pollen stimuli of bee‐pollinated plants (Roulston & Cane, 2000). Yellow and UV‐absorbing pigments like quercetin determine the color of pollen grains of many flowering plants (Lunau, 1995, 2000; Stanley & Linskens, 1985). Eugenol is a common floral volatile and has been shown to be emitted by pollen of bee‐pollinated plants (Dobson & Bergström, 2000; Dobson, Bergström, & Groth, 1990; Dobson et al., 1999). The free amino acid proline is a candidate gustatory key stimulus for pollen (Carter, Shafir, Vaknin, Palmer, & Thornburg, 2006; Wacht, Lunau, & Hansen, 1996), since it is a very common constituent in the pollenkitt of many angiosperms (Lehmann, Funck, Szabados, & Rentsch, 2010; Schmidt & Hanna, 2006; Stanley & Linskens, 1974). Experienced as well as inexperienced honeybees showed a proboscis reaction upon antennal contact with natural pollen (Arenas & Farina, 2012; Grüter, Arenas, & Farina, 2008). In order to test whether sugars might also improve the attractiveness of pollen, the monosaccharide glucose was chosen as a stimulus. Glucose is an essential constituent of floral nectar (Wykes, 1952), but is also found in pollen (Nepi, Guarnieri, & Pacini, 2003; Roulston & Cane, 2000). Recently, it was shown that bumblebees also collect pollen surrogates and even accept pollen surrogates that are inert and without any nutritive value such as cellulose powder or glass pellets (Konzmann & Lunau, 2014; Lunau et al., 2015). To simulate the tactile properties of pollen masses, pollen‐grain‐sized glass pellets were taken as a stimulus. The glass pellets of a diameter 40‐80 μm match the size of common pollen grains of entomophilous flowers (Harder & Thomson, 1989). In this study, we explicitly tested whether pollen displays visual, olfactory, gustatory, and tactile key stimuli that enable bees to detect and identify pollen.

To analyze the behavioral sequence of the tested bumblebee workers to the presented stimuli, the experiments were recorded by a high‐speed camera and analyzed according to distinct behavioral reactions which were: approach, antennal contact, landing, proboscis extension, buzzing and biting with mandibles. Thus, by using an experimental setup, we provide insights into how bumblebees respond to uni‐ and multimodal pollen stimuli, which is a first step toward an understanding of pollen as salient objects and not only as nutritious reward.

The experiments were carried out at variable room temperature from July 2012 to March 2014 in an indoor bumblebee laboratory at the Institute of Sensory Ecology of the Heinrich‐Heine‐University Düsseldorf. According to the bumblebees activity, more experiments were conducted in the morning than in the afternoon. For behavioral experiments, we used queenright colonies of *Bombus terrestris* (re‐natur GmbH, Ruhwinkel, Germany) with approximately 20–40 individual worker bees. The hive was connected by a corridor to a general flight cage (80 cm high, 40 cm long, 40 cm wide), where the bumblebees were allowed to forage on the nectar surrogate Biogluc^®^ diluted with tab water in 1:1 ratio which was offered in 50‐ml tubes. The bumblebees had access to a separate experimental flight cage (20 cm high, 30 cm long, 30 cm wide) by a corridor made of Plexiglas, which could be locked during experimental trials to make sure that the test was performed by a single worker only. Two fluorescent tubes (L58W/865, Lumilux cool daylight, Osram, Munich, Germany) illuminated the experimental flight cage between 8 a.m. and 8 p.m. To analyze the behavioral sequence of the tested bumblebee workers to the presented stimuli, the experiments were recorded using a high‐speed camera and analyzed according to distinct behavioral reactions which were approach, antennal contact, landing, proboscis extension, buzzing and biting with mandibles. The image section of the camera (Panasonic HC‐V707, Kadoma, Japan) was focused on the center of the single artificial flower tested at a time and one individual of *Bombus terrestris* was released into the experimental flight cage. During the first approach to the artificial flower, shooting was started and the activity of the tested bumblebee was recorded for one minute. All responses of each individual bee within the 1 min time interval were evaluated. Afterward. the bumblebee was captured, tagged, and released into the general flight cage. The experiment was terminated if the bumblebee did not respond within 5 min; non‐responding bumblebees were excluded from the analysis. The artificial flower was changed and a new trial was started with another bumblebee.

### 2.2 Test stimuli

Artificial flowers consisted of a circular filter paper (Rundfilter NM 615, Ø 70 mm, Machery‐Nagel, Düren, Germany) to which three test stimuli were attached. Before each test the artificial flower was fixed by means of a steel wire vertically at the inner wall of the experimental flight cage. For the preparation of the test stimuli, small circular disks with a diameter of 5.5 mm were cut out of filter paper (Rundfilter MN615; Macherey‐Nagel, Düren, Germany) with a perforator and were treated with the test stimuli (Table 1). Between one and five stimuli were applied to the small disks as follows: 13.1 mg glass pellets (Worf‐Glasskugeln Ø 40–80 μm) were transferred with forceps and fixed with glue (Methylan^®^ by Henkel, Germany) onto the disk. This glue is pure methylcellulose and thus has no smell; the solvent control also contained methylcellulose and never attracted bumblebees. 10 μl quercetin powder suspended in distilled water (quercetin‐dihydrate, 97%, Alfa Aeser^®^; normal concentration 3.357 × 10^−5^ mol/ml; high concentration 3.357 × 10^−4^ mol/ml) was transferred with a pipette onto the disk. The volume of 2 μl L‐proline (Merck^®^, Darmstadt; 1.3897 × 10^−4^ mol/ml in destilled water), 10 μl glucose‐monohydrate (Merck^®^, Darmstadt; 8.8810 × 10^−4^ mol/ml in destilled water), and 10 μl eugenol (Merck^®^, Darmstadt; 6.5164 × 10^−5^ mol/ml in n‐hexane) were added using a pipette. After the application of one substance, the disk was dried for some minutes until the next substance was applied. Gustatory stimuli were applied last in order to avoid overlaying effects of other substances. To avoid transmission of odors or other contaminants, gloves were used while handling the artificial flower.

**Table 1:** Stimuli tested in various combinations

Substance	Supplier	Effect	Amount applied to disk
Quercetin‐dihydrate	Alfa Aeser^®^	Visual	10 μl of 3.6 × 10^−5^ mol/ml in distilled water
Eugenol	NORMAPUR^®^	Olfactory	10 μl of 6.5 × 10^−5^ mol/ml solution in hexane
Glucose‐monohydrate	Merck	Gustatory	10 μl of 8.9 × 10^−4^ mol/ml in distilled water
L‐Proline	Merck	Gustatory	2 μl of 1.4 × 10^−4^ mol/ml in distilled water
Glass pellets	Worf Glaskugeln	Tactile	13.1 mg (40–80 μm), methylcellulose paste

Dandelion pollen (*Taraxacum officinale*) was collected in the months from April to August 2014 and was fixed to the small filter paper disk with glue (Methylan^®^ by Henkel, Germany). The plants were picked in the botanical garden of the Heinrich‐Heine‐University, Düsseldorf, Germany prior to pollen presentation. After they were kept for 1 day in containers with water, the fresh pollen was extracted, and preserved in the refrigerator. Dandelion pollen was chosen because the plants are known to have a long flowering period and thus fresh pollen was available throughout the experimental testing. Dandelion pollen is collected by bumblebees as a major protein source (Teper, 2006; Whittington, Winston, Tucker, & Parachnowitsch, 2004), although bumblebees cannot live on pure dandelion pollen diet (Génissel, Aupinel, Bressac, Tasei, & Chevrier, 2002). The diameter of dandelion pollen grains is about 25–30 μm.

All solvents used to prepare the stimulus combination were applied to the small filter paper disk of the solvent control.

**Figure 1:**
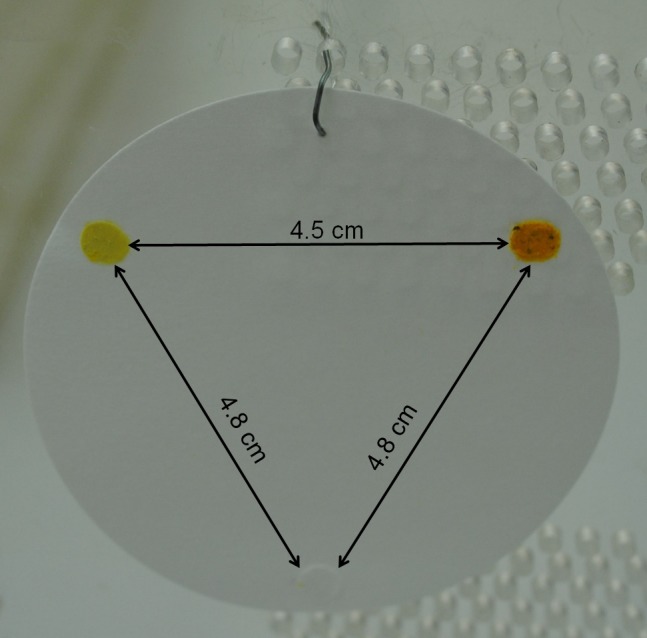
Example of the presentation of the test stimuli on the filter paper with a stimulus combination including quercetin (top left), natural dandelion pollen (top right) and the solvent control (bottom). Distances indicated for explanation

Three test stimuli—stimulus combination, pollen, and solvent control—were fixed with glue (Methylan^®^ by Henkel, Germany) to a circular filter paper (Rundfilter MN615; Ø 70 mm, Macherey‐Nagel) in a distance of about 45 mm from each other (Figure 1). For each test series a minimum of twelve round filters were prepared each with three prepared disks, resp. test stimuli, and used as replicates. These three disks were fixed in all possible spatial arrangements to avoid position effects. All spatial arrangements were offered in equal numbers in the tests.

### 2.3 Experimental procedure

The spontaneous behavior of single flower‐naive bumblebees was tested using a single artificial flower displaying the triple choice test. The bumblebees were not trained before the test trial.

The experiments were performed between 8 a.m. and 4 p.m. Only one flower‐naive individual at a time was released in the experimental flight cage and its behavior was recorded by a camera, Casio Exilim Ex‐F1 equipped with a 36–432‐mm objective. High‐speed videos were recorded by this digital camera, positioned in an angle of 20° to the surface of the artificial flower and outside of the test cage, so that the activities of the bumblebees were clearly visible on the frames. To distinguish bumblebees that had already been tested from experimentally naive ones, the animals were tagged after the experimental trial with a numbered label (Ophalithplättchen, Holtermann, Brockel, Germany). All workers were naive and used only for one test.

The videos were used to categorize the behavioral reactions, which were defined as follows:

An *approach* is classified if the bumblebee approaches unequivocally one of the three test stimuli and targets at it by directing the flagellum of its antennae at the test stimulus distance and the antennal tips being closer than 1 cm. The distance between antennal tips and target stimulus was estimated by using the threefold length of the flagellum.
An *antennae reaction* is classified if the bumblebee`s antennae are in physical contact with the artificial flower after an approach.
A *landing* is classified if all 6 legs of the bumblebee are in physical contact with the artificial flower after an antennal reaction.

Proboscis extension, mandible biting, and buzzing were exhibited only rarely and in an irregular sequence.

The position of the test stimuli, uni‐/multimodal stimulus combination, natural dandelion pollen, and solvent control were changed with each test to exclude the effect of position preferences of the bumblebees. A test series ended when twelve tested bumblebees showed at least one reaction. In three cases we tested 24 instead of 12 bumblebees.

With help of the video, the behavioral reactions of individual bumblebee workers were classified and indexed as follows.

$$\begin{array}{c} \text{Index of behavior}\;\left(\text{IB}\right)=\frac{\text{Number of reactions to stimulus}}{\left(\text{Number of reactions to stimulus}+\text{Number of reactions to dandelion pollen}\right)} \end{array}$$

Index of behavior (IB) is a parameter with values between 0 and 1. IB < 0.5 indicates that the frequency of bumblebees’ reactions to the dandelion pollen is higher than that to the tested stimulus combination. IB = 0.5 means bumblebees reacted to pollen and to stimulus combination equally often. IB > 0.5 indicates that the frequency of bumblebees’ reactions to the tested stimulus combination (with 1–5 stimuli) surpasses that of the dandelion pollen. Reactions toward the solvent control were never observed and thus are not included in the calculation of IB. The median value of IB calculated for all bumblebee workers (*n* = 12 or 24) that responded to the same stimulus was calculated. IB values for all bumblebee workers that responded to the same stimulus were tested against 0.5 (i.e., equal number of responses to stimulus and dandelion pollen) using a Wilcoxon signed‐rank test. In addition, we corrected for multiple tests using the false discovery rate (Benjamini & Hochberg, 1995). Significant deviations from 0.5 indicate either preferences for dandelion pollen (IB < 0.5) or stimuli (> 0.5). All statistical analyses were performed using the statistical software R (R Core Team 2015).

## 3 Results

**Figure 2:**
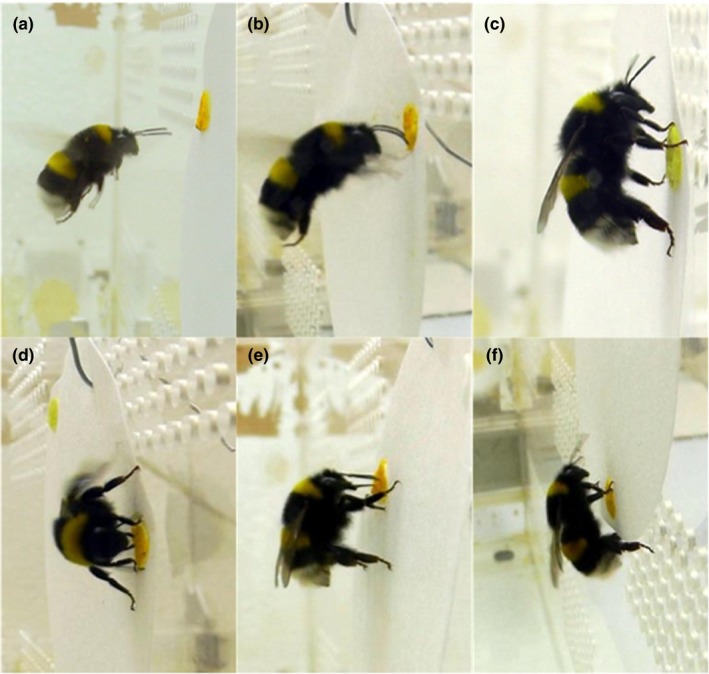
Classification and evaluation of recorded behavioral reactions: a approach, alignment of antennae toward the artificial flower, pollen or treatment, from close distance; b antennae reaction, touching the surface of the stimulus with antennae; c landing, touching the flower dummy with all legs; d mandible reaction, visible mandible agitation; e proboscis reaction, visible proboscis extension; f buzzing, audible vibration of flight musculature

The tested bumblebees regularly exhibited approaches, antenna reactions and landings at one or two of the three close‐range stimuli presented at a single disk (Figure 2). The bumblebees never responded to the solvent control and thus the solvent control was not used to calculate the IB. In total, 773 approaches of bumblebee workers toward the dandelion pollen were registered. 652 (84.3%) of these approaches ended up with an antenna reaction, and 517 (66.9%) with a landing reaction. Relatively few approaches led to proboscis extension (5.0%), mandible biting (6.0%), and/or buzzing (4.9%). The bumblebees approached the various stimulus combinations 183 times. 73.2% of these approaches ended up with antennal reaction, 56.8% with a landing reaction. In only 0.5% the bumblebees approach led to a proboscis extension, in 1.1% to mandible biting, and in 0.5% to buzzing (Figure 2).

The number of bumblebees that responded to dandelion pollen as constant stimulus in all tests was high and consistent: 11.04 ± 1.07 and a minimum of 9 out of 12 tested workers in one test series approached the dandelion pollen (Table S1); 10.82 ± 1.26 and a minimum of 8 workers antennated at it (Table S2), and 9.64 ± 1.92 and a minimum of 5 workers landed on it during the test interval of 1 min (Table S3). Proboscis extension, mandible biting, and buzzing were only rarely observed (Tables S4–S6).

**Figure 3:**
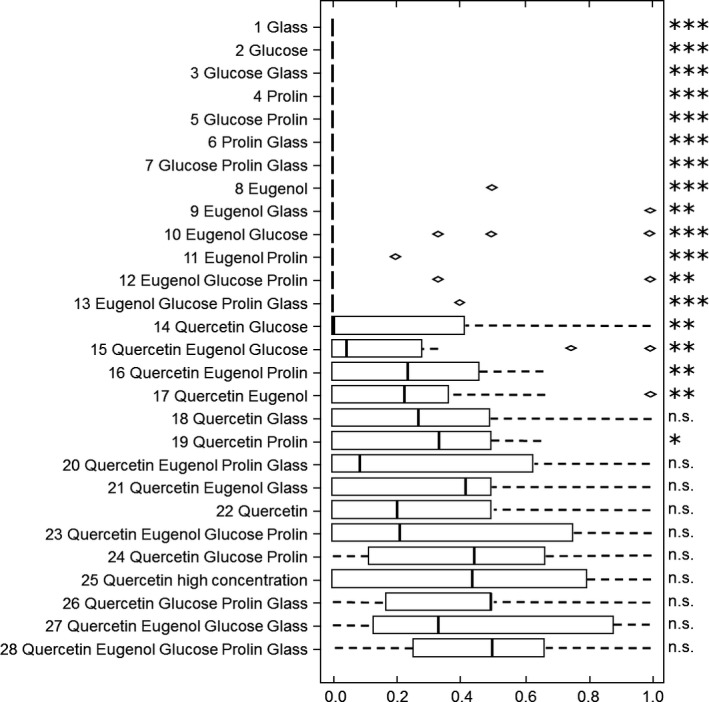
Preference during approach of Bombus terrestris workers for the tested stimulus combinations, based on the index of behavior (IB = 0.5: no preference; IB < 0.5: preference for pollen over stimulus combination; IB > 0.5: preference for stimulus combination over pollen). Indicated are median, quartiles, whiskers, and outliers. A paired Wilcoxon test was applied if *n* ≥ 6 to test for differences in the index of behavior for pollen and stimulus combination. Level of significance: *p* ≤ .001 ≙ ***p ≤ .01 ≙ **p ≤ .05 ≙ *, n.s. ≙ not significant. Results that do not remain significant after correction for multiple tests (FDR, Benjamini & Hochberg, 1995) are shown in bracts

In contrast, the bumblebees approached, antennated, and landed at the stimulus combination only if quercetin was present excluding some exceptions. In the 13 experiments in which the stimulus combination did not include quercetin, the bumblebees approached the stimulus combination in 1.2% of all responses, whereas 98.8% of the approaches were directed to the dandelion pollen. In those 15 experiments, in which the stimulus combination included quercetin, the bumblebees approached the stimulus combination in 31.0% of all responses, and 69.0% of the approaches were directed to the dandelion pollen (Figure 3). When quercetin was present in the stimulus combination 7.47 ± 1.31 and a minimum of five individuals approached the stimulus combination (Table S1), 6.13 ± 1.93 and a minimum of two bumblebees antennated at it (Table S2), 5.07 ± 1.84 and a minimum of one bumblebee landed on it within the experimental test interval (Table S3).

For the most complete stimulus combination, including quercetin, eugenol, glucose, proline, and glass, the number of bumblebees responding at least once was as high as for dandelion pollen with regard to approach, antennal reaction, and landing, and further reactions such as buzzing, proboscis extension, and mandible biting (Tables S4–S6).

**Figure 4:**
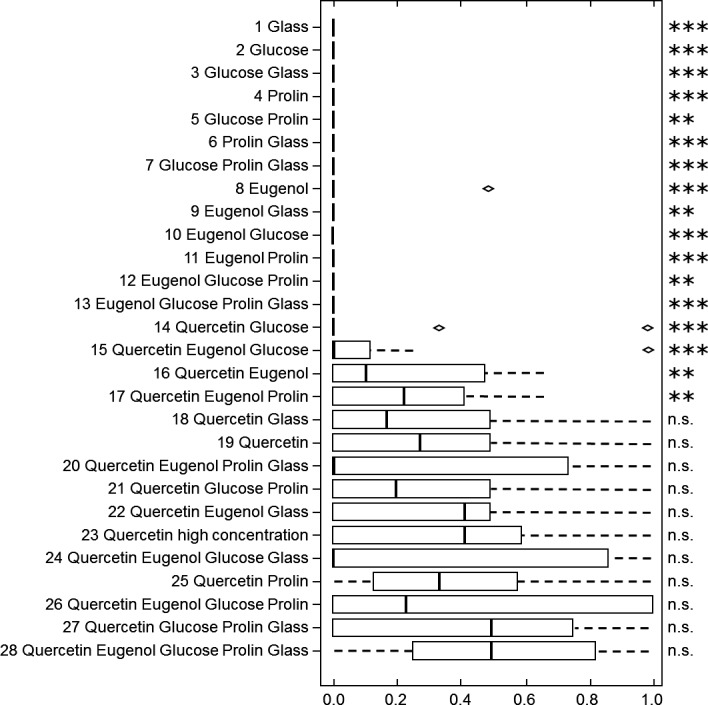
Preference during antennal reaction of Bombus terrestris workers for the tested stimulus combinations, based on the index of behavior (IB = 0.5: no preference; IB < 0.5: preference for pollen over stimulus combination; IB > 0.5: preference for stimulus combination over pollen). Detailed information is given in the legend of Figure 3. A paired Wilcoxon test was applied if *n* ≥ 6 to test for differences in the index of behavior for pollen and stimulus combination. Level of significance: *p* ≤ .001 ≙ ***p ≤ .01 ≙ **p ≤ .05 ≙ *, n.s. ≙ not significant. Results that do not remain significant after correction for multiple tests (FDR, Benjamini & Hochberg, 1995) are shown in bracts

**Figure 5:**
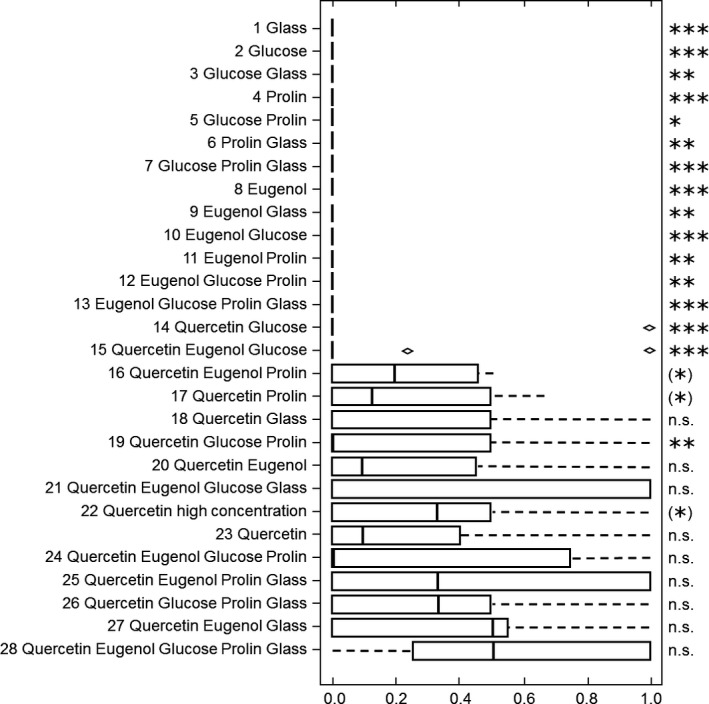
Preference during landing of Bombus terrestris workers for the tested stimulus combinations, based on the index of behavior (IB = 0.5: no preference; IB < 0.5: preference for pollen over stimulus combination; IB > 0.5: preference for stimulus combination over pollen). Detailed information is given in the legend of Figure 3. A paired Wilcoxon test was applied if *n* ≥ 6 to test for differences in the index of behavior for pollen and stimulus combination. Level of significance: *p* ≤ .001 ≙ ***p ≤ .01 ≙ **p ≤ .05 ≙ *, n.s. ≙ not significant. Results that do not remain significant after correction for multiple tests (FDR, Benjamini & Hochberg, 1995) are shown in bracts

The bumblebees’ further responses were evaluated for those tests, in which the stimulus combination included the visual stimulus quercetin: The portion of approaches that were followed by an antennal reaction was not significantly different for approaches toward dandelion pollen (84.3%) and approaches toward the stimulus combinations including quercetin (73.2%; Fisher's exact test, *p* = .295; Figure 4). Moreover, the portion of antennal reactions that ended up with a landing was also not significantly different for antennal reactions at dandelion pollen (79.3%) and antennal reactions at the stimulus combinations including quercetin (77.6%; Fisher's Exact test, *p* = .940; Figure 5). However, significantly more landings on dandelion pollen as compared to landings on the stimulus combinations including quercetin were followed by mandible biting, proboscis extension or buzzing 24.8%, resp. 3.8% (Fisher's exact test, *p* < .001).

No general correlation was found for the portion of antennal reactions that ended up with a landing and the number of tactile or chemical stimuli presented at stimulus combinations including quercetin (*r* = −.013; *p* = .965; Pearson). However, the number of stimuli combined in a stimulus combination seemingly predicts the frequency of responses of the bumblebees toward the stimulus combination. In only 4 experiments (17: Que Eug Pro; 22: Que Eug Gla; 26: Que Eug Glu Pro; 28: Que Eug Glu Pro Gla) did the bumblebees antennate more often at the test stimulus combination (containing quercetin) following an approach when compared to dandelion pollen; all these four stimulus combinations contained eugenol (Figure 4, Tables S2 and S7). In 6 (15: Que Eug Glu; 20: Que Eug; 24: Que Eug Glu Pro; 25: Que Eug Pro Gla; 27: Que Eug Gla; 28: Que Eug Glu Pro Gla) out of eight experiments, in which the stimulus combination (containing quercetin) contained eugenol, the portion of antennal reactions that ended up with a landing was larger for the stimulus combination than for the dandelion pollen (Figure 5, Tables S3 and S7). Moreover, these were the only stimulus combinations for which the portion of antennal reactions that ended up with a landing was larger for the stimulus combination than for the dandelion pollen (Figure 5, Table S7).

## 4 Discussion

Our experimental results demonstrate that the common pollen pigment quercetin is necessary and sufficient to elicit a spontaneous full behavioral response in previously inexperienced bumblebees including approach, antennal reaction and landing, confirming that visual signals are highly important to trigger innate responses to pollen signals (Lunau, 1992, 2000). In some experiments the bumblebees responded equally or even better to the stimulus combination than to the natural dandelion pollen indicating attractive stimuli in the stimulus combination. Additional stimuli thus might facilitate pollen detection and increase the frequency of subsequent behavioral reactions such as antennal contact and landing, whereas the bumblebees did not respond to olfactory stimuli alone, emphasizing the efficacy of multimodal stimuli for pollen detection and recognition. These results indicate that multimodal signals are not only important for flower recognition (Junker & Parachnowitsch, 2015), but also for pollen recognition in naive bumblebees. The importance of interactions between floral traits of different sensory modalities for various flower visitors including bees have been emphasized by Junker and Parachnowitsch (2015) and Rüdenauer et al. (2015). For experienced bees, it has been demonstrated that pollen odor strongly affects the bees’ foraging behavior (Cook et al., 2005).

The results of this study strengthen the view that multimodal stimuli are responsible for triggering full behavioral responses to pollen in bumblebees, which is particularly evident in behavioral reactions following the visually guided approach. For the decision to land on a flower, the visual stimulus alone is sufficient, but additional stimuli of other modalities increase the frequency of landings. Of note, a higher concentration of the pigment quercetin also resulted in an increase in approaches suggesting that the salience of the visual stimulus (compare to Katzenberger et al., 2013) in the normal stimulus combinations was suboptimal; however, the difference in the frequency of approaches toward the normal and the tenfold concentration of quercetin was not significant. UV‐absorbing yellow is by the far the most common pollen color in plants and caused by flavonoid pigments known for antifungal and antibacterial properties and shielding against the mutagenic UV light (Lunau, 1995, 2000; Osche, 1979; Pacini & Hesse, 2005). However, preference tests with bumblebees suggest that the UV‐absorbing yellow color hue is not the decisive color parameter triggering the bumblebees’ antennal response (Lunau, Wacht, & Chittka, 1996; Rohde, Papiorek, & Lunau, 2013). There is, however, evidence that visual pollen cues play a dominant role in pollen detection by bees. Bumblebees visiting natural flowers exhibit the antennal reaction not only at stamens, but also at stamen‐mimicking floral guides (Lunau, 1991, 1992). Stamen‐ and pollen‐mimicking floral guides are a very common feature of bee‐pollinated flowers and mostly display a UV‐absorbing yellow color (Lunau, 2000, 2006) that is contrasting against the rest of the flower (Lunau, 1992). Conspicuous pollen‐ and stamen‐mimicking floral guides are more likely to attract bees for antennal contact if combined with less attractive, that is, not UV‐absorbing and yellow, real pollen and stamens (Lunau, 2006).

In our experiments, chemical and tactile stimulants of the stimulus combinations slightly increase the frequency of decision for closer inspection of the stimulus combinations. It is known that pollenkitt is a solvent for chemical pollen stimuli, but neither a common pollen odor (Dobson, 1988; Dobson & Bergström, 2000) nor a typical pollen taste has been described (Pacini & Hesse, 2005). An exception is the free amino acid proline which is a common constituent of the pollenkitt of angiosperm pollen grains (Lehmann et al., 2010; Stanley & Linskens, 1974; Weiner, Hilpert, Werner, Linsenmair, & Blüthgen, 2010) and thought to support pollen germination and pollen tube growth due to its hygroscopic property (Britikov & Musatova, 1964; Dashek, 1970). Due to its widespread presence in pollenkitt, proline would represent a suitable key stimulus for pollen detection. Electrophysiological studies with the labellar and tarsal taste bristles of the pollen‐feeding syrphid fly *Eristalis tenax* have shown that the salt receptor in these flies is much more sensitive to the amino acid proline than to cations and might be regarded as a proline receptor (Wacht, Lunau, & Hansen, 2000). It is unlikely that proline is detected prior to contact due to its poor olfactory detectability (Linander, de Ibarra, & Laska, 2012).

There is little experimental evidence that tactile cues might be important for triggering distinct behavioral responses in pollen‐collecting insects. Bees are known to collect chemically inert pollen surrogates such as glass powder or cellulose powder from emasculated or artificial flowers (Burkart, Schlindwein, & Lunau, 2014; Konzmann & Lunau, 2014).

Relatively few bumblebees tried to harvest the pollen by extending the proboscis, biting with the mandibles or by buzzing. They exhibited these behavioral reactions only rarely, but significantly more often at the natural dandelion pollen as compared to the stimulus combination. Only the stimulus combinations including all tested stimuli, namely quercetin, eugenol, glucose, proline, and glass powder, elicited as many responses as the natural pollen; this was valid for all reactions such as approach, antennation, landing, and subsequent reactions. This result may be interpreted that additive effects of various stimuli contributing to the attractiveness of pollen and stimulus combinations. Lunau et al. (2015) found conflicting evidence for key stimuli eliciting pollen collection in bumblebees, which readily collected chemically inert pure glass powder, stopped collecting pollen if it was embittered by quinine obviously only after probing pollen eventually. Some flowers hide their pollen grains poricidal anthers and bees buzz these flowers even if the anther pores have been glued indicating that they are obviously unable to detect visual or chemical pollen signals before buzzing the flowers, but immediately recognize pollen release (Burkart et al., 2014).

Testing bumblebees which were naive in regard to pollen would have improved this study. However, the bumblebees’ experience with natural pollen cannot be excluded, because all bumblebees feed on pollen as larvae and experience pollen odor in the nest. By feeding pollen in the dark nest, the premature conditioning of the yellow pollen color could be avoided.

The presented data suggest that multimodal pollen stimuli are effectively stimulating pollen collection behavior in bumblebees and that a visual color stimulus seems essential to start the behavioral sequence. The hierarchy of multimodal cues is seemingly linked to the sequence of behavioral reactions, initiated by the visually guided approach and continued by olfactory, gustatory, and tactile stimuli triggering subsequent reactions. Multimodal stimulus combinations were able to trigger bumblebees’ responses as strong as natural dandelion pollen. Although the chemical stimuli tested in this study might be rather common among pollen, none of eugenol, proline, or sucrose is known as key substance for bees to identify pollen (Dobson & Bergström, 2000). It remains open, how a somehow arbitrary, but multimodal combination of a small number of stimuli, can trigger responses as strong as natural pollen, but it seems likely that addressing several sensory modalities, that is, a multimodal stimulus, is important to trigger pollen seeking behavior.

## Conflict of Interest

None declared.
