# Supplementary material for: Bumblebees require visual pollen stimuli to initiate and multimodal stimuli to complete a full behavioral sequence in close‐range flower orientation
Source: Ecol Evol. 2017 Feb 1;7(5):1384–93. doi: 10.1002/ece3.2768 (PMC5357828; doi:10.1002/ece3.2768)
Supplement: Supplementary file 1 [file ECE3-7-1384-s001.docx]

**Supporting information**

Tab. S1: Number of approaching bumblebees exhibiting an absolute preference for the dandelion pollen (IB=0), an absolute preference for the stimulus combination (IB=1), an intermediate preference (IB<1;IB>0), or showed no response.

| **Approach** | **Preference for pollen** | **Preference for stimulus comb** | **Intermediate preference** | **No response** |
| --- | --- | --- | --- | --- |
|  | *IB = 0* | *IB = 1* | *IB < 1 & IB > 0* | *IB = n/a* |
| Eugenol | 22 | 0 | 1 | 0 |
| Glucose Proline | 12 | 0 | 0 | 0 |
| Proline Glass | 12 | 0 | 0 | 0 |
| Glucose Glass | 12 | 0 | 0 | 0 |
| Glucose Proline Glass | 12 | 0 | 0 | 0 |
| Eugenol Glucose | 22 | 0 | 2 | 0 |
| Eugenol Proline | 11 | 0 | 1 | 0 |
| Eugenol Glass | 11 | 1 | 0 | 0 |
| Eugenol Glucose Proline | 10 | 1 | 1 | 0 |
| Eugenol Glucose Proline Glass | 11 | 0 | 1 | 0 |
| Quercetin | 10 | 6 | 8 | 0 |
| Quercetin high concentration | 4 | 3 | 5 | 0 |
| Quercetin Glass | 5 | 1 | 6 | 0 |
| Quercetin Glucose Proline | 2 | 2 | 8 | 0 |
| Quercetin Glucose Proline Glass | 3 | 2 | 7 | 0 |
| Quercetin Eugenol Glass | 5 | 1 | 6 | 0 |
| Quercetin Eugenol Glucose Proline | 5 | 3 | 4 | 0 |
| Quercetin Eugenol Glucose Glass | 3 | 3 | 6 | 0 |
| Quercetin Eugenol Proline Glass | 6 | 2 | 4 | 0 |
| Quercetin Eugenol Glucose Proline Glass | 3 | 2 | 7 | 0 |
| Glass | 12 | 0 | 0 | 0 |
| Glucose | 12 | 0 | 0 | 0 |
| Proline | 12 | 0 | 0 | 0 |
| Quercetin Glucose | 7 | 1 | 4 | 0 |
| Quercetin Eugenol Glucose | 6 | 1 | 5 | 0 |
| Quercetin Eugenol | 5 | 1 | 6 | 0 |
| Quercetin Eugenol Proline | 5 | 0 | 7 | 0 |
| Quercetin Proline | 4 | 0 | 8 | 0 |

Tab. S2: Number of antennating bumblebees exhibiting an absolute preference for the dandelion pollen (IB=0), an absolute preference for the stimulus combination (IB=1), an intermediate preference (IB<1;IB>0), or showed no response.

| **Antennation** | **Preference for pollen** | **Preference for stimulus comb** | **Intermediate preference** | **No response** |
| --- | --- | --- | --- | --- |
|  | *IB = 0* | *IB = 1* | *IB < 1 & IB > 0* | *IB = n/a* |
| Eugenol | 23 | 0 | 0 | 0 |
| Glucose Proline | 11 | 0 | 0 | 1 |
| Proline Glass | 12 | 0 | 0 | 0 |
| Glucose Glass | 12 | 0 | 0 | 0 |
| Glucose Proline Glass | 12 | 0 | 0 | 0 |
| Eugenol Glucose | 24 | 0 | 0 | 0 |
| Eugenol Proline | 12 | 0 | 0 | 0 |
| Eugenol Glass | 11 | 0 | 0 | 1 |
| Eugenol Glucose Proline | 11 | 0 | 0 | 1 |
| Eugenol Glucose Proline Glass | 12 | 0 | 0 | 0 |
| Quercetin | 11 | 5 | 7 | 1 |
| Quercetin high concentration | 3 | 2 | 5 | 2 |
| Quercetin Glass | 6 | 1 | 5 | 0 |
| Quercetin Glucose Proline | 4 | 2 | 6 | 0 |
| Quercetin Glucose Proline Glass | 4 | 2 | 6 | 0 |
| Quercetin Eugenol Glass | 5 | 1 | 6 | 0 |
| Quercetin Eugenol Glucose Proline | 5 | 4 | 3 | 0 |
| Quercetin Eugenol Glucose Glass | 7 | 3 | 2 | 0 |
| Quercetin Eugenol Proline Glass | 7 | 3 | 2 | 0 |
| Quercetin Eugenol Glucose Proline Glass | 3 | 3 | 6 | 0 |
| Glass | 12 | 0 | 0 | 0 |
| Glucose | 12 | 0 | 0 | 0 |
| Proline | 12 | 0 | 0 | 0 |
| Quercetin Glucose | 10 | 1 | 1 | 0 |
| Quercetin Eugenol Glucose | 10 | 1 | 1 | 0 |
| Quercetin Eugenol | 6 | 1 | 5 | 0 |
| Quercetin Eugenol Proline | 5 | 0 | 7 | 0 |
| Quercetin Proline | 5 | 1 | 6 | 0 |

Tab. S3: Number of landing bumblebees exhibiting an absolute preference for the dandelion pollen (IB=0), an absolute preference for the stimulus combination (IB=1), an intermediate preference (IB<1;IB>0), or showed no response.

| **Landing** | **Preference for pollen** | **Preference for stimulus comb** | **Intermediate preference** | **No response** |
| --- | --- | --- | --- | --- |
|  | *IB = 0* | *IB = 1* | *IB < 1 & IB > 0* | *IB = n/a* |
| Eugenol | 11 | 0 | 0 | 1 |
| Glucose Proline | 7 | 0 | 0 | 5 |
| Proline Glass | 7 | 0 | 0 | 5 |
| Glucose Glass | 9 | 0 | 0 | 3 |
| Glucose Proline Glass | 12 | 0 | 0 | 0 |
| Eugenol Glucose | 11,5 | 0 | 0 | 1 |
| Eugenol Proline | 9 | 0 | 0 | 3 |
| Eugenol Glass | 11 | 0 | 0 | 1 |
| Eugenol Glucose Proline | 8 | 0 | 0 | 4 |
| Eugenol Glucose Proline Glass | 11 | 0 | 0 | 1 |
| Quercetin | 6,5 | 2,5 | 2,5 | 1 |
| Quercetin high concentration | 3 | 1 | 5 | 3 |
| Quercetin Glass | 7 | 1 | 4 | 0 |
| Quercetin Glucose Proline | 7 | 2 | 3 | 0 |
| Quercetin Glucose Proline Glass | 5 | 2 | 4 | 1 |
| Quercetin Eugenol Glass | 5 | 1 | 6 | 0 |
| Quercetin Eugenol Glucose Proline | 6 | 3 | 2 | 1 |
| Quercetin Eugenol Glucose Glass | 5 | 3 | 1 | 3 |
| Quercetin Eugenol Proline Glass | 4 | 3 | 1 | 4 |
| Quercetin Eugenol Glucose Proline Glass | 3 | 4 | 5 | 0 |
| Glass | 12 | 0 | 0 | 0 |
| Glucose | 12 | 0 | 0 | 0 |
| Proline | 12 | 0 | 0 | 0 |
| Quercetin Glucose | 10 | 1 | 0 | 1 |
| Quercetin Eugenol Glucose | 8 | 1 | 1 | 2 |
| Quercetin Eugenol | 6 | 1 | 5 | 0 |
| Quercetin Eugenol Proline | 6 | 0 | 6 | 0 |
| Quercetin Proline | 5 | 0 | 5 | 2 |

Tab. S4: Number of proboscis extending bumblebees exhibiting an absolute preference for the dandelion pollen (IB=0), an absolute preference for the stimulus combination (IB=1), an intermediate preference (IB<1;IB>0), or showed no response.

| **Proboscis extension** | **Preference for pollen** | **Preference for stimulus comb** | **Intermediate preference** | **No response** |
| --- | --- | --- | --- | --- |
|  | *IB = 0* | *IB = 1* | *IB < 1 & IB > 0* | *IB = n/a* |
| Eugenol | 7 | 0 | 0 | 16 |
| Glucose Proline | 1 | 0 | 0 | 11 |
| Proline Glass | 1 | 0 | 0 | 11 |
| Glucose Glass | 2 | 0 | 0 | 10 |
| Glucose Proline Glass | 3 | 0 | 0 | 9 |
| Eugenol Glucose | 2 | 0 | 0 | 22 |
| Eugenol Proline | 0 | 0 | 0 | 12 |
| Eugenol Glass | 1 | 0 | 0 | 11 |
| Eugenol Glucose Proline | 2 | 0 | 0 | 10 |
| Eugenol Glucose Proline Glass | 0 | 0 | 0 | 12 |
| Quercetin | 2 | 0 | 0 | 22 |
| Quercetin high concentration | 1 | 0 | 0 | 11 |
| Quercetin Glass | 1 | 0 | 0 | 11 |
| Quercetin Glucose Proline | 0 | 1 | 0 | 11 |
| Quercetin Glucose Proline Glass | 2 | 0 | 0 | 10 |
| Quercetin Eugenol Glass | 2 | 0 | 0 | 10 |
| Quercetin Eugenol Glucose Proline | 0 | 0 | 0 | 12 |
| Quercetin Eugenol Glucose Glass | 1 | 0 | 0 | 11 |
| Quercetin Eugenol Proline Glass | 0 | 0 | 0 | 12 |
| Quercetin Eugenol Glucose Proline Glass | 1 | 0 | 0 | 11 |
| Glass | 0 | 0 | 0 | 12 |
| Glucose | 1 | 0 | 0 | 11 |
| Proline | 4 | 0 | 0 | 8 |
| Quercetin Glucose | 0 | 0 | 0 | 12 |
| Quercetin Eugenol Glucose | 2 | 0 | 0 | 10 |
| Quercetin Eugenol | 0 | 0 | 0 | 12 |
| Quercetin Eugenol Proline | 2 | 0 | 0 | 10 |
| Quercetin Proline | 3 | 0 | 0 | 9 |

Tab. S5: Number of mandible biting bumblebees exhibiting an absolute preference for the dandelion pollen (IB=0), an absolute preference for the stimulus combination (IB=1), an intermediate preference (IB<1;IB>0), or showed no response.

| **Mandible biting** | **Preference for pollen** | **Preference for stimulus comb** | **Intermediate preference** | **No response** |
| --- | --- | --- | --- | --- |
|  | *IB = 0* | *IB = 1* | *IB < 1 & IB > 0* | *IB = n/a* |
| Eugenol | 2 | 0 | 0 | 21 |
| Glucose Proline | 0 | 0 | 0 | 12 |
| Proline Glass | 0 | 0 | 0 | 12 |
| Glucose Glass | 1 | 0 | 0 | 11 |
| Glucose Proline Glass | 0 | 0 | 0 | 12 |
| Eugenol Glucose | 2 | 0 | 0 | 22 |
| Eugenol Proline | 1 | 0 | 0 | 11 |
| Eugenol Glass | 1 | 0 | 0 | 11 |
| Eugenol Glucose Proline | 0 | 0 | 0 | 12 |
| Eugenol Glucose Proline Glass | 1 | 0 | 0 | 11 |
| Quercetin | 3 | 0 | 0 | 21 |
| Quercetin high concentration | 0 | 0 | 0 | 12 |
| Quercetin Glass | 2 | 0 | 0 | 10 |
| Quercetin Glucose Proline | 3 | 0 | 0 | 9 |
| Quercetin Glucose Proline Glass | 0 | 0 | 0 | 12 |
| Quercetin Eugenol Glass | 0 | 0 | 0 | 12 |
| Quercetin Eugenol Glucose Proline | 0 | 0 | 1 | 11 |
| Quercetin Eugenol Glucose Glass | 0 | 0 | 0 | 12 |
| Quercetin Eugenol Proline Glass | 0 | 0 | 0 | 12 |
| Quercetin Eugenol Glucose Proline Glass | 0 | 1 | 0 | 11 |
| Glass | 5 | 0 | 0 | 7 |
| Glucose | 1 | 0 | 0 | 11 |
| Proline | 2 | 0 | 0 | 10 |
| Quercetin Glucose | 2 | 0 | 0 | 10 |
| Quercetin Eugenol Glucose | 2 | 0 | 0 | 10 |
| Quercetin Eugenol | 3 | 0 | 0 | 9 |
| Quercetin Eugenol Proline | 3 | 0 | 0 | 9 |
| Quercetin Proline | 3 | 0 | 0 | 9 |

Tab. S6: Number of buzzing bumblebees exhibiting an absolute preference for the dandelion pollen (IB=0), an absolute preference for the stimulus combination (IB=1), an intermediate preference (IB<1;IB>0), or showed no response.

| **Buzzing** | **Preference for pollen** | **Preference for stimulus comb** | **Intermediate preference** | **No response** |
| --- | --- | --- | --- | --- |
|  | *IB = 0* | *IB = 1* | *IB < 1 & IB > 0* | *IB = n/a* |
| Eugenol | 0 | 0 | 0 | 23 |
| Glucose Proline | 0 | 0 | 0 | 12 |
| Proline Glass | 0 | 0 | 0 | 12 |
| Glucose Glass | 0 | 0 | 0 | 12 |
| Glucose Proline Glass | 0 | 0 | 0 | 12 |
| Eugenol Glucose | 2 | 0 | 0 | 22 |
| Eugenol Proline | 0 | 0 | 0 | 12 |
| Eugenol Glass | 0 | 0 | 0 | 12 |
| Eugenol Glucose Proline | 0 | 0 | 0 | 12 |
| Eugenol Glucose Proline Glass | 0 | 0 | 0 | 12 |
| Quercetin | 1 | 0 | 0 | 23 |
| Quercetin high concentration | 0 | 0 | 0 | 12 |
| Quercetin Glass | 0 | 0 | 0 | 12 |
| Quercetin Glucose Proline | 1 | 0 | 0 | 11 |
| Quercetin Glucose Proline Glass | 0 | 0 | 0 | 12 |
| Quercetin Eugenol Glass | 0 | 0 | 0 | 12 |
| Quercetin Eugenol Glucose Proline | 0 | 0 | 0 | 12 |
| Quercetin Eugenol Glucose Glass | 0 | 0 | 0 | 12 |
| Quercetin Eugenol Proline Glass | 0 | 0 | 0 | 12 |
| Quercetin Eugenol Glucose Proline Glass | 0 | 1 | 0 | 11 |
| Glass | 5 | 0 | 0 | 7 |
| Glucose | 0 | 0 | 0 | 12 |
| Proline | 0 | 0 | 0 | 12 |
| Quercetin Glucose | 3 | 0 | 0 | 9 |
| Quercetin Eugenol Glucose | 2 | 0 | 0 | 10 |
| Quercetin Eugenol | 2 | 0 | 0 | 10 |
| Quercetin Eugenol Proline | 2 | 0 | 0 | 10 |
| Quercetin Proline | 3 | 0 | 0 | 9 |

Tab. S7: Number of behavioral reactions (approach, antennal reaction, landing, proboscis extension, mandible biting, buzzing) at the dandelion pollen and at the stimulus combination.

| **Number of behavioural reactions (n) at dandelion pollen** | | | | | | |
| --- | --- | --- | --- | --- | --- | --- |
|  | approach | antennae | landing | proboscis | mandibels | buzzing |
| Eugenol Glass | 30 | 26 | 23 | 1 | 1 | 0 |
| Eugenol Glucose Proline Glass | 30 | 26 | 19 | 0 | 1 | 0 |
| Eugenol Glucose Proline | 20 | 18 | 11 | 2 | 0 | 0 |
| Eugenol Glucose | 35 | 26 | 25 | 0 | 0 | 0 |
| Eugenol Proline | 37 | 30 | 24 | 0 | 1 | 0 |
| Eugenol | 22 | 18 | 17 | 3 | 0 | 0 |
| Glucose Glass | 25 | 23 | 13 | 2 | 1 | 0 |
| Glucose Proline Glass | 28 | 16 | 14 | 4 | 0 | 0 |
| Glucose Proline | 17 | 14 | 7 | 1 | 0 | 0 |
| Proline Glass | 24 | 22 | 8 | 1 | 0 | 0 |
| Quercetin Eugenol Glass | 22 | 20 | 14 | 2 | 0 | 0 |
| Quercetin Eugenol Glucose Glass | 27 | 21 | 13 | 1 | 0 | 0 |
| Quercetin Eugenol Glucose Proline Glass | 14 | 11 | 10 | 1 | 0 | 0 |
| Quercetin Eugenol Glucose Proline | 20 | 16 | 11 | 0 | 1 | 0 |
| Quercetin Eugenol Proline Glass | 30 | 17 | 6 | 0 | 0 | 0 |
| Quercetin Glass | 29 | 24 | 18 | 1 | 2 | 0 |
| Quercetin Glucose Proline Glass | 15 | 12 | 10 | 2 | 0 | 0 |
| Quercetin Glucose Proline | 36 | 35 | 31 | 0 | 8 | 6 |
| Quercetin highly concentrated | 21 | 19 | 17 | 1 | 0 | 0 |
| Quercetin | 27 | 20 | 16 | 0 | 1 | 0 |
| Glass | 45 | 42 | 36 | 0 | 7 | 9 |
| Glucose | 28 | 22 | 22 | 1 | 3 | 0 |
| Proline | 34 | 29 | 29 | 6 | 2 | 0 |
| Quercetin Eugenol Glucose | 37 | 33 | 27 | 3 | 4 | 3 |
| Quercetin Eugenol Proline | 31 | 31 | 29 | 3 | 8 | 6 |
| Quercetin Eugenol | 34 | 33 | 30 | 3 | 3 | 5 |
| Quercetin Glucose | 32 | 27 | 19 | 0 | 4 | 5 |
| Quercetin Proline | 23 | 21 | 18 | 4 | 4 | 4 |
|  |  |  |  |  |  |  |
| **total** | **773** | **652** | **517** | **42** | **51** | **38** |
|  | | | | | | |
| **Number of behavioural reactions (n) at the stimulus combination** | | | | | | |
|  | approach | antennae | landing | proboscis | mandibels | buzzing |
| Eugenol Glass | 1 | 0 | 0 | 0 | 0 | 0 |
| Eugenol Glucose Proline Glass | 2 | 0 | 0 | 0 | 0 | 0 |
| Eugenol Glucose Proline | 2 | 0 | 0 | 0 | 0 | 0 |
| Eugenol Glucose | 0 | 0 | 0 | 0 | 0 | 0 |
| Eugenol Proline | 1 | 0 | 0 | 0 | 0 | 0 |
| Eugenol | 0 | 0 | 0 | 0 | 0 | 0 |
| Glucose Glass | 0 | 0 | 0 | 0 | 0 | 0 |
| Glucose Proline Glass | 0 | 0 | 0 | 0 | 0 | 0 |
| Glucose Proline | 0 | 0 | 0 | 0 | 0 | 0 |
| Proline Glass | 0 | 0 | 0 | 0 | 0 | 0 |
| Quercetin Eugenol Glass | 13 | 13 | 13 | 0 | 0 | 0 |
| Quercetin Eugenol Glucose Glass | 18 | 12 | 7 | 0 | 0 | 0 |
| Quercetin Eugenol Glucose Proline Glass | 12 | 11 | 11 | 0 | 1 | 1 |
| Quercetin Eugenol Glucose Proline | 10 | 10 | 7 | 0 | 1 | 0 |
| Quercetin Eugenol Proline Glass | 11 | 7 | 6 | 0 | 0 | 0 |
| Quercetin Glass | 11 | 9 | 7 | 0 | 0 | 0 |
| Quercetin Glucose Proline Glass | 14 | 11 | 8 | 0 | 0 | 0 |
| Quercetin Glucose Proline | 17 | 10 | 5 | 1 | 0 | 0 |
| Quercetin highly concentrated | 13 | 10 | 8 | 0 | 0 | 0 |
| Quercetin | 7 | 6 | 3 | 0 | 0 | 0 |
| Glass | 0 | 0 | 0 | 0 | 0 | 0 |
| Glucose | 0 | 0 | 0 | 0 | 0 | 0 |
| Proline | 0 | 0 | 0 | 0 | 0 | 0 |
| Quercetin Eugenol Glucose | 9 | 3 | 3 | 0 | 0 | 0 |
| Quercetin Eugenol Proline | 14 | 11 | 8 | 0 | 0 | 0 |
| Quercetin Eugenol | 11 | 10 | 10 | 0 | 0 | 0 |
| Quercetin Glucose | 6 | 2 | 1 | 0 | 0 | 0 |
| Quercetin Proline | 11 | 9 | 7 | 0 | 0 | 0 |
|  |  |  |  |  |  |  |
| **total** | **183** | **134** | **104** | **1** | **2** | **1** |
